# Women Overestimate Temporal Duration: Evidence from Chinese Emotional Words

**DOI:** 10.3389/fpsyg.2017.00004

**Published:** 2017-01-18

**Authors:** Mingming Zhang, Lingcong Zhang, Yibing Yu, Tiantian Liu, Wenbo Luo

**Affiliations:** ^1^Research Center of Brain and Cognitive Neuroscience, Liaoning Normal UniversityDalian, China; ^2^Department of Psychology, Minnan Normal UniversityZhangzhou, China; ^3^Laboratory of Cognition and Mental Health, Chongqing University of Arts and SciencesChongqing, China

**Keywords:** emotional word, language processing, time perception, duration estimation, gender differences

## Abstract

Numerous studies have proven the effect of emotion on temporal perception, using various emotional stimuli. However, research investigating this issue from the lexico-semantic perspective and gender difference remains scarce. In this study, participants were presented with different types of emotional words designed in classic temporal bisection tasks. In Experiment 1 where the arousal level of emotional words was controlled, no pure effect of valence on temporal perception was found; however, we observed the overestimation of women relative to men. Furthermore, in Experiment 2, an orthogonal design of valence and arousal with neutral condition was employed to study the arousal-mechanism of temporal distortion effect and its difference between genders. The results showed that the gender difference observed in Experiment 1 was robust and was not influenced by valence and arousal. Taken together, our findings suggest a stable gender difference in the temporal perception of semantic stimuli, which might be related to some intrinsic properties of linguistic stimuli and sex differences in brain structure as well as physiological features. The automatic processing of time information was also discussed.

## Introduction

In the recent decade, a large number of studies have revealed the effects of emotion on temporal perception to varying degrees, by employing facial expressions (Droit-Volet et al., 2004; Zhang et al., 2014b; Li and Yuen, 2015), emotional situations (Lui et al., 2011; Gil and Droit-Volet, 2012; Grondin et al., 2014), musical emotions (Noulhiane et al., 2007; Smith et al., 2011; Droit-Volet et al., 2013; Schirmer et al., 2016), bodily expressions (Droit-Volet and Gil, 2015), and even emotional colors (Shibasaki and Masataka, 2014) or odors (Millot et al., 2016; Yue et al., 2016). It should be noted that the evidence is positioned within the perspectives in models of scalar expectancy theory, which identify the requirement of an arousal-attention mechanism to interpret such an emotional time distortion effect (Gibbon, 1977; Gibbon et al., 1984). To be specific, firstly, increasing arousal accelerates the pacemaker rate; subsequently, more pulses are received by the accumulator, and longer duration is observed-that is, a temporal dilation effect and vice versa. Secondly, when the attentional resources demanded for timing processing are impaired, underestimation of duration will occur.

However, to our knowledge, no study has investigated the emotional time distortion effect of emotionally laden words, despite a growing body of literature focused in the research field of emotion and time. In addition, some studies on emotion seemingly tended to emphasize that non-verbal materials could induce stronger emotional effect than words because of their direct biological cues (Kissler et al., 2006) and higher visual complexity (Schlochtermeier et al., 2013). In fact, emotional words, as highly symbolic lexis, also play an important role in our daily life and social communication, similar to other emotional stimuli, especially for the reading. For instance, Kousta et al. (2009) presented a series of random strings and asked the participants to judge whether they were true or false words. They found that emotional words could be perceived faster and more accurately than neutral words regardless of polarity (Kousta et al., 2009). Concerning the translation of text, in an interesting study of Hsu et al. (2015), 24 German second language learners were required to read short passages from the popular Harry Potter books in two versions (i.e., German and English), implying negative, positive, or neutral valence. Their results revealed that, compared to reading in a second language, the emotional reading of native language could elicit stronger activation of bilateral amygdala and the left precentral cortex for “happy” than “neutral” (Hsu et al., 2015).

Furthermore, some research findings have preliminarily reported the time course of neural dynamics of emotional stimuli, using the event-related potential technique, which indicated that the neural processing of emotional adjectives also consisted of three stages (i.e., automatic processing, distinguishing emotional and neutral information, and emotion separation, successively; Zhang et al., 2014a), which is similar with facial expressions (Luo et al., 2010), emotional pictures (Zhu et al., 2015), and nouns (Yi et al., 2015). This similarity inherently centers around a fundamental fact that the resulting distributions of various emotional materials share an underlying two-dimensional affective space (Bradley and Lang, 1999; Bradley et al., 2001a). In addition, with the literature review on electrophysiological and hemodynamic neuroimaging methodologies in the last 10 years, Citron (2012) concluded that emotionally relevant words could elicit cortical or cerebral reactions qualitatively, which is comparable to pictures and faces.

In summary, based on the above-mentioned similarities, the present study expects to reveal the effect of emotion on temporal perception at a lexico-semantic level. Thus, in Experiment 1, we selected emotional words with controlled arousal level to investigate the pure effect of valence (negative, neutral, and positive). In Experiment 2, we further manipulated the other one dimension of affective space (i.e., arousal) to examine whether the arousal-mechanism of emotional time distortion still exists in the semantic symbol.

On the other hand and interestingly, we found that numerous corresponding studies were specific to women only (e.g., Droit-Volet et al., 2004; Lui et al., 2011; Droit-Volet and Gil, 2015), or had dominant woman participation (e.g., Smith et al., 2011; Droit-Volet et al., 2013; Tamm et al., 2014). Even though the overall woman-to-man ratio in some experiments was 0.5, the gender difference of emotional time distortion was neither examined nor reported (e.g., Gil and Droit-Volet, 2011b; Droit-Volet et al., 2015). Nevertheless, gender constitutes an important factor that influences cognitive processes and emotional reactivity (Hyde, 2014). For example, Bradley et al. (2001a) proposed that human-beings’ emotional reactions were composed of defensive motivation and appetitive motivation, and sex differences affect motivational activation (Bradley et al., 2001b). Morever, a number of studies on behavior or neuroscience have documented that women possess the following gender-specific traits: better ability of emotion recognition, emotional memory, and increased susceptibility to negative stimuli (LaBar and Cabeza, 2006; Collignon et al., 2010).

Evidence also exists that the sex effects on elementary time processing tend to be small, but relatively stable (for a review, see Block et al., 2000). These studies are multifaceted and range from seconds (e.g., Rammsayer and Rammstedt, 2000; Hancock and Rausch, 2010; Glicksohn and Hadad, 2012; Bartholomew et al., 2015) to minutes (e.g., Espinosa-Fernández et al., 2003) or even to our lifespan (e.g., Hancock, 2010; Hancock and Hancock, 2013). With respect to the short duration examined in our study (i.e., from 400 to 1600 ms), generally, women make a larger time estimation than men, although there are various research methodologies in temporal perception (Hornstein and Rotter, 1969).

Therefore, we postulate that the emotional time distortion effect is modulated by the gender factor. In Experiments 1 and 2, we will investigate the common effect of gender and emotion on temporal perception.

## Experiment 1

### Method

#### Participants

As paid volunteers, 28 healthy right-handed individuals (age = 19.61 ± 0.96; 13 men) with normal or corrected-to-normal vision participated in the experiment after giving their written informed consents. The study was approved by Liaoning Normal University Human Research Institutional Review Board in accordance with the Declaration of Helsinki (1991).

#### Materials

Considering that adjectives usually describe characteristics, states or traits and may be related more directly to emotions than nouns and verbs (Palazova et al., 2011), emotional adjectives were used in this study to investigate the pure effect of emotional valence on temporal perception.

The stimuli consisted of 30 Chinese adjectives (10 negative, 10 neutral, and 10 positive ones; see Appendix Table A1), excluding words referring to speed (Zhang et al., 2014), that were selected from the Chinese Affective Words System (CAWS, containing total 1500 emotional two-character words selected from the Modern Chinese Dictionary of Commonly Used Words. These words were rated by 124 participants in valence, arousal, and dominance on a 9-point scale; Wang et al., 2008). All three types of adjectives appeared with similar frequencies^1^ [*F*(2,27) = 0.50, *p* > 0.05, $\eta_{p}^{2}$ = 0.035; negative: *M* = 107.60, *SD* = 104.17, neutral: *M* = 147.10, *SD* = 121.82, positive: *M* = 109.50, *SD* = 66.27], strokes [*F*(2,27) = 1.04, *p* > 0.05, $\eta_{p}^{2}$ = 0.071; negative: *M* = 19.30, *SD* = 4.88, neutral: *M* = 17.00, *SD* = 2.75, positive: *M* = 16.90, *SD* = 4.31], and fixed arousal [*F*(2,27) = 0.01, *p* > 0.05, $\eta_{p}^{2}$ < 0.001; negative: *M* = 4.86, *SD* = 0.17, neutral: *M* = 4.85, *SD* = 0.49, positive: *M* = 4.91, *SD* = 0.39] while they differed significantly in valence [*F*(2,27) = 721.76, *p* < 0.001, $\eta_{p}^{2}$ = 0.982; negative: *M* = 2.91, *SD* = 0.16, neutral: *M* = 5.03, *SD* = 0.33, positive: *M* = 6.89, *SD* = 0.18]. The font of their characters was Song Ti No.48. All stimuli were presented in white on the black background with the same contrast, brightness and size of 142 pixels × 68 pixels. The screen resolution was 60 pixels per inch. Subjects were seated in a dimly lit and sound-proof room with their eyes approximately 70 cm from a 19-inch screen. All stimuli were displayed in the center of the screen.

#### Procedure

**FIGURE 1:**
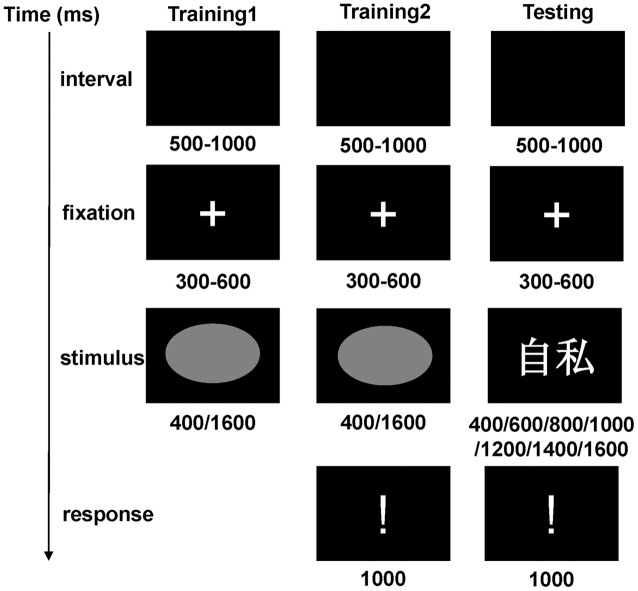
**A depiction of the training 1, training 2, and testing phases in the temporal bisection task**.

The temporal bisection task is one of the most frequently employed procedures when investigating the effects of emotion on temporal perception (Droit-Volet et al., 2004; Droit-Volet and Gil, 2015). Everyone received three successive phases: two trainings and one testing (**Figure 1**). The experimental procedure was programmed and recorded with E-Prime 2.0 software (Psychology Software Tools, Inc.).

In the first training phase, participants were trained to know the “Short” and “Long” standard durations well. Each trial started with a random 500–1000 ms blank screen, and a 300–600 ms fixation cross then appeared in the center of screen. Immediately, “Short” or “Long” standard durations in the form of a gray oval (12 cm × 16 cm) was displayed for 400 or 1600 ms, respectively. Each standard was presented five times in alternation which was counterbalanced. At the end of this phase, we asked participants whether they noticed the difference between these two standards.

In the second training phase, the order of these two standard durations was randomized (four times for each condition). Each trial started with presentation of a gray oval (400 or 1600 ms) followed by an exclamation point. Participants needed to discriminate whether the stimulus’s duration was “Short” or “Long” by pressing one of two computer keys (“F” or “J”). Responses with latencies less than 1000 ms were considered valid. The right or wrong feedback would be presented and the response key was counterbalanced across participants. All participants reached 100% accuracy in their performance before starting the test session.

After those training blocks, subjects were given the testing phase. They were presented with negative, neutral, or positive adjectives in a series of comparison durations (400/600/800/1000/1200/1400/1600 ms); feedback was discontinued. They were then instructed to indicate whether the presentation duration of the stimulus was more similar to the “Short” or to the “Long” standard by pressing the corresponding key (“F” or “J”). The assignment of keys to “F” and “J” responses was counterbalanced across participants as well.

A total of 210 trials (30 words × 7 durations) were randomly assigned to three blocks, in which each word-duration pair was displayed only once. Standard stimuli (“Short” and “Long” ovals) were presented five times each at the beginning of each block to prevent participants from forgetting them. Blocks were separated by self-terminated breaks. All blocks and trials within each block were presented in a random order.

### Results and Discussion

The proportion of “Long” of each individual from each emotional valence condition was fit with a pseudo-logistic model (**Formula 1**; Killeen et al., 1997; Allan, 2002; Droit-Volet and Gil, 2015), using the GraphPad Prism^®^ 6 software. This model fitted the data of Experiment 1 well, mean *R*^2^ = 0.919, *SE* = 0.019.

$$f(x)=1/\left[1+\exp\left((\omega -x)/\left(\sqrt{3\gamma x}/\pi \right)\right)\right]$$

In **Formula 1**, the proportionality constant ω is the bisection point [BP; the point of subjective equality, which indicates the stimulus durations for which the *p* (Long) = 0.5] and _γ_ is the Weber Ratio (WR; an index of time sensitivity, lower WR, greater sensitivity to time). Statistical analyses were based on the temporal dilation effect (TD; i.e., 1000 ms minus BP, a positive TD means temporal overestimation and vice versa) and WR.

**FIGURE 2:**
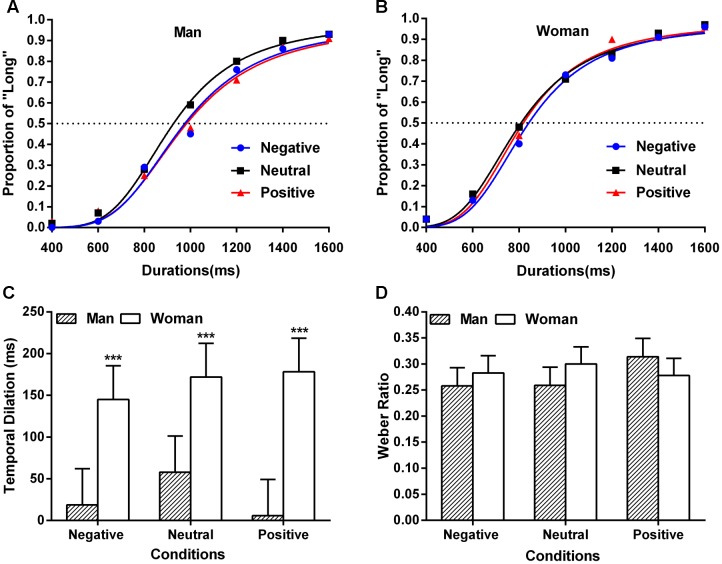
**(A)** Mean proportion of “Long” responses and the fit of the psychometric functions in negative, neutral, and positive emotional adjectives with the stimuli duration of ranging from 400 to 1600 ms for the man. **(B)** Mean proportion of “Long” responses and the fit of the psychometric functions in negative, neutral, and positive emotional adjectives with the stimuli duration of ranging from 400 to 1600 ms for the woman. The dotted line means the 50% of “Long” responses. **(C)** Results of the temporal distortion effect from Experiment 1 for different valence and gender. ^∗∗∗^*p* < 0.001 in a one-sample *t*-test against 0. **(D)** Results of the WR for different valence and gender. Error bars show standard errors. See also the **Supplementary Tables S1**–**S3**.

Average psychometric functions estimated from the Prism accordingly for men and women are shown in **Figures 2A,B**. The analysis of variance (ANOVA) on the fitting *R*^2^ with valence type and gender as factors did not yield any significant effect (*p*s > 0.05).

The TD was tested with an ANOVA for the factors emotional valence and gender, and a significant main effect of gender was observed [*F*(1,78) = 16.26, *p* < 0.001, $\eta_{p}^{2}$ = 0.173]. The TD in women (*M* = 165 ms, *SE* = 23 ms) was larger than that in men (*M* = 28 ms, *SE* = 25 ms). The main effect of valence type and their interactive effect were not significant (*p*s > 0.05). The results of one-sample *t*-test showed that the TD in women [*t*(14)s > 4.17, *p*s < 0.001] rather than men [*t*(12)s < 1, *p*s > 0.05] was significantly different from zero for each condition (**Figure 2C**), suggesting that only women overestimated the length of duration presented for each emotional condition at the semantic level.

The index of time sensitivity (i.e., WR) was tested with an ANOVA for emotional valence and gender (**Figure 2D**). No significant effect or interaction was found (*p*s > 0.05), suggesting that the time discrimination was not affected by neither emotional valence nor gender.

In summary, the findings from Experiment 1 revealed that there was only the gender effect, and not an emotional valence effect, on temporal perception based on the semantic level. That is, women overestimated time regardless of the valence of emotional adjectives, but this phenomenon did not occur in men. In Experiment 1, we only employed moderate arousal adjectives. Thus, we further performed Experiment 2 to investigate those questions: when arousal rating changes, (1) whether the arousal-mechanism of emotional time distortion would occur, (2) whether the gender difference on temporal perception observed still exists.

## Experiment 2

### Methods

#### Participants

As paid volunteers, 26 healthy right-handed individuals (age = 19.35 ± 1.16; 13 men) with normal or corrected-to-normal vision participated in the experiment after giving their written informed consents. This study was approved by Liaoning Normal University Human Research Institutional Review Board in accordance with the Declaration of Helsinki (1991).

#### Materials

The stimuli consisted of 50 Chinese adjectives (10 high-arousal positive, 10 high-arousal negative, 10 low-arousal positive, 10 low-arousal negative, and 10 neutral ones used in Experiment 1^2^; see Appendix Table A2), not containing some words describing different speeds (Zhang et al., 2014), that were selected from the CAWS. Relevant characteristics of these adjectives are depicted in **Table 1**. The ANOVA performed on valence rating revealed a significant main effect of valence type [*F*(2,47) = 1122.56, *p* < 0.001, $\eta_{p}^{2}$ = 0.979; positive or negative vs. neutral: *p*s < 0.001]. The ANOVA on arousal rating revealed a significant main effect of arousal type [*F*(2,47) = 175.69, *p* < 0.001, $\eta_{p}^{2}$ = 0.882]. In addition, comparing the stimuli in Experiment 2 with the ones used in Experiment 1 then showed that there was no significant difference in the strokes [*F*(7,72) = 0.704, *p* = 0.669, $\eta_{p}^{2}$ = 0.064] and in the occurrence frequencies [*F*(7,72) = 0.183, *p* = 0.988, $\eta_{p}^{2}$ = 0.017] across all conditions. Meanwhile, the valence of negative adjectives in Experiment 2 did not differ from those in Experiment 1 significantly [*t*(28) = 1.45, *p* > 0.05] and the valence of positive adjectives in Experiment 2 did not differ from those in Experiment 1 significantly as well [*t*(28) = 0.86, *p* > 0.05]. All in all, these primary factors considered in Experiment 2 were manipulated and counterbalanced well.

**Table 1:** Descriptive statistics for selected words in Experiment 2 (*M* ± *SD*).

	Low-Arousal Negative	High-Arousal Negative	Low-Arousal Positive	High-Arousal Positive	Neutral
Valence	3.10 ± 0.20	3.01 ± 0.11	6.94 ± 0.21	6.97 ± 0.20	5.03 ± 0.33
Arousal	4.14 ± 0.22	5.97 ± 0.29	4.03 ± 0.26	5.86 ± 0.22	4.86 ± 0.49
Stroke	19.10 ± 5.53	17.40 ± 3.92	16.10 ± 2.64	18.50 ± 4.72	17.00 ± 2.75
Frequency	108.70 ± 106.84	133.40 ± 194.43	122.00 ± 172.32	118.30 ± 138.85	147.10 ± 121.82


#### Procedure

The stimuli presentation and the task were similar to those in Experiment 1 without the followings. Because of the addition of arousal, the trials of testing phase increased to 350 (50 adjectives × 7 durations) and were divided into five blocks, in which each word-duration pair was displayed only once. As in Experiment 1, the standard stimuli (“Short” and “Long” ovals) were also presented five times each at the beginning of each block to prevent the participants from forgetting them.

### Results and Discussion

**FIGURE 3:**
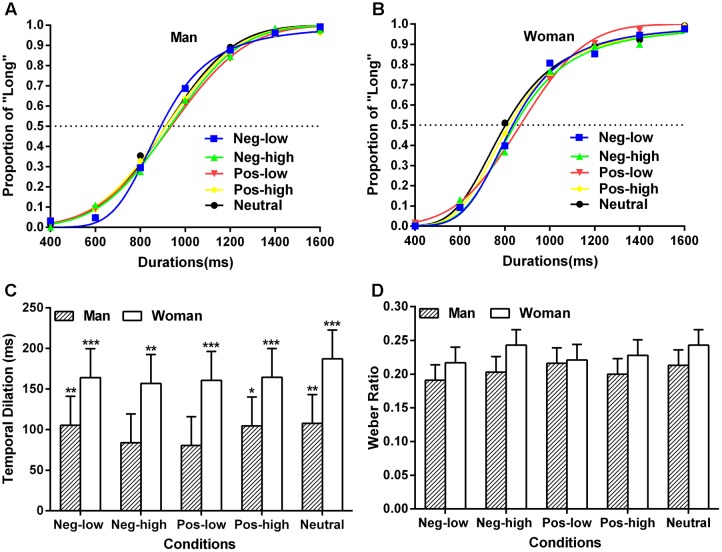
**(A)** Mean proportion of “Long” responses and the fit of the psychometric functions in each condition with the stimuli duration of ranging from 400 to 1600 ms for the man. **(B)** Mean proportion of “Long” responses and the fit of the psychometric functions in each condition with the stimuli duration of ranging from 400 to 1600 ms for the woman. The dotted line means the 50% of “Long” responses. **(C)** Results of the temporal distortion effect from Experiment 2 for different condition and gender. ^∗^*p* < 0.05, ^∗∗^*p* < 0.01, ^∗∗∗^*p* < 0.001 in a one-sample *t*-test against 0. **(D)** Results of the WR for different condition and gender. Error bars show standard errors. See also the **Supplementary Tables S4**–**S6**.

The average psychometric functions estimated from the Prism for men and women were shown in **Figures 3A,B** separately. As a while, this pseudo-logistic model fitted the data of Experiment 2 well, mean *R*^2^ = 0.970, *SE* = 0.008. The ANOVA on the fitting *R*^2^ with emotional conditions and gender as factors did not display any significant effects (*p*s > 0.05).

The TD was measured by ANOVA of emotional conditions (high-arousal positive, high-arousal negative, low-arousal positive, low-arousal negative, and neutral) × gender, and a robust significant main effect of gender was also observed [*F*(1,120) = 9.78, *p* = 0.002, $\eta_{p}^{2}$ = 0.075]. The TD in women (*M* = 167 ms, *SE* = 15 ms) was larger than that in men (*M* = 96 ms, *SE* = 15 ms). The main effect of emotional conditions and their interactive effect were not significant (*p*s > 0.05). Furthermore, for women, the results of one-sample *t*-test showed that the TD was significantly different from 0 when they perceived each type of emotional words [*t*(12)s > 4.02, *p*s < 0.01] (**Figure 3C**), suggesting that women always overestimated the length of durations presented irrespective of specific content at the semantic level. Similar analysis was conducted for men, which demonstrated that the significant differences between TD and 0 emerged in negative low-arousal [*t*(12) = 3.30, *p* < 0.01], positive high-arousal [*t*(12) = 2.52, *p* < 0.05], and neutral items [*t*(12) = 3.21, *p* < 0.01]. The results of same statistical analysis in negative high-arousal [*t*(12) = 2.13, *p* = 0.054] and positive low-arousal words [*t*(12) = 1.95, *p* = 0.075] both approached significance marginally (**Figure 3C**).

We also tested a three way ANOVA (valence × arousal × gender) for the TD performance, which just found a significant main effect of gender [*F*(1,96) = 6.98, *p* = 0.010, $\eta_{p}^{2}$ = 0.068]. The other main effects and interactive effects were not significant (*p*s > 0.05).

The WR for different gender and five conditions was illustrated in **Figure 3D**. There was not any significant effect found in this same ANOVA for the index of time sensitivity (*p*s > 0.05). A supplementary three way ANOVA analysis (valence × arousal × gender) for the WR could not also yield any significant results (*p*s > 0.05). These findings indicated that the time discrimination was not disrupted by emotional adjective type and gender, and that these task difficulties were similar between different conditions.

In summary, the results of Experiment 2 revealed that when arousal rating changes, the robust gender difference on temporal perception still exists (i.e., the overestimation performance of women compared to men), however, which appeared somewhat different from this in Experiment 1. Specifically, at a semantic level, the temporal lengthening effect of women might not be influenced by emotional arousal both in these two experiments while men only overestimated the durations of words presented in Experiment 2 in which the emotional arousal increased or decreased as opposed to these in Experiment 1, although some performance of men only reached marginal statistical significance. In addition, the classical arousal-mechanism of emotional time distortion did not occur in all participants.

## General Discussion

The present study aimed to investigate the gender-differentiated effect of emotional time distortion at a semantic level using emotional adjectives, which was based mainly on the following consideration derived from previous studies: Emotional words and other emotional stimuli share the similar affective space pattern and processing characteristics, as mentioned in the section “Introduction.”

In Experiment 1, we only manipulated the emotional valence and fixed the arousal rating, but no pure effect of valence was observed on temporal perception. A series of studies demonstrated that temporal judgments can also be influenced by emotional valence, especially for facial expressions in which a temporal overestimation generally occur in expressions of anger, fear, happiness, and sadness, while inverse effect emerges in ashamed faces, and no temporal distortion occurs in disgusted expressions (for a review, see Gil and Droit-Volet, 2011a). However, the present results are most consistent with those of previous studies in the demonstration that valence did not affect duration estimates of emotional images (e.g., Van Volkinburg and Balsam, 2014).

In Experiment 2, we changed the emotional arousal of negative and positive adjectives in order to investigate whether the arousal-mechanism of emotional time distortion would occur, that is, increasing arousal would elicit a temporal lengthening effect (Gibbon, 1977; Gibbon et al., 1984). Unfortunately, this classical performance proved by numerous studies could not be found in the present research. On the one hand, the findings might be interpreted within some intrinsic properties of linguistic stimuli as opposed to those that are emotional, despite those similarities described in the section “Introduction.” For instance, differences in the picture and word processing were discussed so extensively in the past decades, which was not understood fully. However, there is a common opinion that pictures are decoded or processed more speedily and superiorly relative to verbal stimuli (e.g., Seifert, 1997; Azizian et al., 2006). This superiority also emerges in the emotions (e.g., Kensinger and Schacter, 2006; Gianotti et al., 2008; Flaisch et al., 2015). Moreover, similar to the methods employed in previous corresponding studies, we used an orthogonal design of valence and arousal with neutral condition as the baseline, which also means that effects of valence and arousal would always appear to be experimentally entangled. In fact, negative words are likely to be much more arousing than positive ones, whereas the frequencies of positive words are much higher than of negative words in most cases, thus suggesting that individuals rate positive words as more familiar (Wang et al., 2008; Citron et al., 2014b). However, to maintain the balance of other attributes of lexical material for each condition that might predict word processing, such as word frequency (Citron, 2012), strokes in a Chinese character, imageability, and concreteness, researchers usually have to make a difficult trade-off among these variables. Consequently, the compromise would finally result in a small difference of arousal rating between negative words and positive emotional words. For example, in the present study, although there is a significantly statistical difference between the arousal levels of emotional words, the essential finding shows that the score of high-arousal and low-arousal approximate at 6 and 4, respectively (from a 9-point scale), which means the observed difference between the arousal levels is not very great.

On the other hand, the disappearance of emotional time distortion effect here might also be associated with the automatic processing of time information. Lewis and Miall (2003)’s review concluded that different time ranges drew upon distinct neural timing systems. The cognitively controlled system depends upon prefrontal and parietal regions for long durations. The automatic system is mainly linked to motor and premotor circuits for short durations, which means that short duration judgments do not need too much attentional resources. Along the same line, this speculation was supported by a growing body of research (e.g., Lewis and Miall, 2003; Buonomano et al., 2009; Morillon et al., 2009; Rammsayer and Ulrich, 2011). Recently, some work of neural oscillation provided a new perspective for this issue (Gu et al., 2015). For instance, Chen et al. (2015) performed a matching-to-sample task and asked participants to estimate and identify whether the durations (1, 2, 3, or 4 s) of two stimuli presented asynchronously were the same, during which electroencephalogram data was recorded. They found the lowest alpha band amplitude in the delay phase of 4-s duration, supporting that short durations (below about 3 s) are encoded as a unit, while longer durations require a memory-based cognitive reconstruction (Fraisse, 1984; Pöppel, 1997). Similarly, the former is relatively automatic, but the latter need more cognitive resources. Consequently, the automatic processing of time information was not disrupted by medium or weak emotions in our study. Further evidence is necessary to investigate the effect of emotional words based on longer durations. Overall, these two considerations might affect the disappearance of arousal-mechanism in emotional time distortion.

Most importantly, the present study showed a reliable gender difference of temporal distortion (compared with men, women always overestimated the durations presented in lexico-semantic level using emotional words), which is, against our predictions, irrelevant to the emotion factor, regardless of valence or arousal. In fact, as early as the beginning of the 20th century, studies by MacDougall (1904) have reported significant sex differences in time estimation, in which a stronger overestimation performance was also found in women than in men. MacDougall’s (1904) research had initiated and spurred subsequent studies on this theme, in which a number of them replicated MacDougall’s findings (Gulliksen, 1927; Goldstone, 1968). However, some did not reveal gender differences in timing (Loehlin, 1959; Roeckelein, 1972; Getsinger, 1974) and some researches even observed contrary results (Harton, 1939). Block et al. (2000) integrated the conflicting results from the methodological perspective and found that using the production technique could obtain consistent pattern. However, our task in the present study differed from their study in some aspects. From one point of view, according to the models of scalar expectancy theory, temporal information processing consists of several cognitive stages, including precise internal clock, memory, and decision process (Gibbon et al., 1984; Rubia and Smith, 2004), which could mean that the categories of cognitive resources (e.g., attention, working memory, updating and inhibition) required by various temporal tasks exist inherent differences because of their specific details (Gil and Droit-Volet, 2011b; Droit-Volet et al., 2015). For instance, some studies emphasized that memory process might play a very limited role in the bisection task because participants could create new reference standards of “Short” and “Long” during the continuous comparison between probe stimuli and original standards (Allan, 2002; Droit-Volet and Rattat, 2007), while this memory capacity is very important to the temporal reproduction task in the same situation (Droit-Volet et al., 2015). Thus, multifarious performances could be observed in previous studies even if the researcher employed the same stimulus material (Gil and Droit-Volet, 2011b). Furthermore, materials used in the present study (i.e., emotional words) not only contain emotional content, but also induce responses with cognitive properties. For example, in our study, after participants were presented with high-arousal negative words (e.g., nasty), they often responded, “This word makes me feel uncomfortable! It’s swearing! It’s immoral!”. That is, specific emotional words might have induced a wider semantic connections and cognitive appraisal (e.g., moral judgment) during timing processing. However, as suggested by Citron (2012) that although a number of studies on emotional words were conducted, the boundary between emotion and cognition remains unclear.

Additionally, the temporal lengthening effect of women relative to men in the present study can be explained by the following considerations. First, to some extent, the larger magnitude of temporal distortion for women indeed reflects the better temporal accuracy of men. According to the primary hypothesis of an oscillating brain mechanism (Grondin, 2001; Rammsayer and Ulrich, 2001), these temporal performances might be caused by the higher neural oscillation speed or greater time resolving ability in male brains. This assumption not only was supported by some behavioral research (Rammsayer and Troche, 2010) but also by neuroanatomical studies. For example, Gur et al. (1999) revealed a larger ratio of white matter compared to gray matter in the male brain. Furthermore, the larger white matter means the faster transmission and processing of cognitive information, that is, a higher neural oscillation as mentioned above. Second, we should consider the physiological differences in both genders. With respect to men, the core body temperature of women is higher across time-of-day (Hancock et al., 1992), which might reflect stronger physical or general cortical arousal that is independent of the influence of emotional stimulation, and then change the frequency of the circadian pacemaker. Hancock (1993) even showed that the increase of 1°C in temperature would be followed by a 10% increase in the speed of temporal estimation. Differences in the rate of functioning of an internal clock between women and men were also discussed by some other authors (Glicksohn and Hadad, 2012).

Furthermore, it has to be noted that men did not generate a temporal distortion effect in Experiment 1 but overestimated the durations of emotional words for each condition presented in Experiment 2, despite the two conditions being marginally significant (negative high-arousal and positive low-arousal). In contrast, the significant women temporal overestimation was observed in both experiments. This observed difference may be ostensibly caused by the change of emotional arousal. However, this point might be explained by the change of trials (i.e., 210 trials in Experiment 1 vs. 350 trials in Experiment 2) rather than by emotional factors that have been found to exert no influence on the present study’s results, because even men had overestimated the durations at low-arousal or neutral levels in Experiment 2. In fact, Hancock and Rausch (2010) also showed similar results, in which, with increasing numbers of trials men’s temporal error estimates increased, while women’s errors remained steady. An accumulative temporal mechanism might account for this difference, which is associated with the accumulation type of Treisman (1963). Evidently, these relevant theories and practices need further exploration.

Finally, one limitation should be discussed here. We did not completely rule out a memory difference between the sexes for the results. In both two experiments, all participants reached 100% accuracy in their performance of training 2 phase before starting the test session and standard stimuli were presented five times each at the beginning of each block to prevent participants from forgetting them, which are consistent with numerous previous studies (e.g., Gan et al., 2009; Zhang et al., 2014b; Droit-Volet et al., 2015). However, the former just can suggest that, in some way, there are no difference between subjects’ resolving ability for the standard durations (i.e., 400 and 1600 ms), and the latter can’t discriminate different memory ability for men and women. Recent review work has highlighted the effect of memory in time perception (Block and Gruber, 2014; Matthews and Meck, 2016). Thus, a further study on this issue should be investigated deeply in the future. Nevertheless, our findings provide some evidence for the sex differences in the processing of semantic time information.

## Conclusion

In summary, this is the first study examining the influence of emotional words on temporal perception as a function of gender. Unfortunately, we did not find any effects of emotional factors (valence or arousal) on time perception. Some intrinsic properties of the linguistic material and the automatic processing of short durations might account for this disparity. In addition, women always overestimated the duration with respect to men regardless the emotional conditions employed in our experiments. This difference may be explainable by sex differences in the brain structure and some physiological features. Finally, for future research, it would be worth investigating whether these effects are dependent on a more detailed classification of emotional words, because some evidence from the literature has indicated that even though the emotional stimuli employed belong to the same category (e.g., fear vs. disgust, both representing threat-related emotions; Zhang et al., 2014b), they have distinct influences on temporal perception. In other words, the impact of the content of experimental material also plays a key role (Gil and Droit-Volet, 2012).

## Ethics Statement

Liaoning Normal University Human Research Institutional Review Board as paid volunteers, all of the subjects with normal or corrected-to-normal vision participated in the experiment after giving their consents. This study was approved by Liaoning Normal University Human Research Institutional Review Board in accordance with the Declaration of Helsinki (1964). All of the subjects here were healthy adults, not containing minors, persons with disabilities or endangered animal species.

## Author Contributions

MZ and WL designed the study. MZ and TL collected and analyzed the data under the supervision of WL and YY. MZ wrote the manuscript. WL, YY, and LZ provided the critical revisions. All authors approved the final version of the manuscript for submission.

## Conflict of Interest Statement

The authors declare that the research was conducted in the absence of any commercial or financial relationships that could be construed as a potential conflict of interest.

## Footnotes

1 The information of frequencies comes from the Dictionary of Usage Frequency of Modern Chinese Words (Liu et al., 1990).

2 On the basis of previous literature using an orthogonal design of valence and arousal with neutral condition as the baseline, we found that the arousal of neutral stimuli always were categorized at a low level regardless of emotional pictures or emotional words (Citron et al., 2014a; Van Volkinburg and Balsam, 2014). However, Experiment 2 used the moderate arousal neutral adjectives (*M* = 4.86, *SD* = 0.49; a 9-point scale) that were consistent with the Experiment 1, by this way, in order to ensure that the arousal effect on temporal perception for negative/positive adjectives was caused by the change of their arousal rating certainly rather than other predictors.
