# Supplementary material for: Women Overestimate Temporal Duration: Evidence from Chinese Emotional Words
Source: Front Psychol. 2017 Jan 18;8:4. doi: 10.3389/fpsyg.2017.00004 (PMC5241309; doi:10.3389/fpsyg.2017.00004)
Supplement: Supplementary file 1 [file Data_Sheet_1.docx]

**Supplementary materials**

**Table S1** Mean proportion of “Long” for different conditions in Experiment 1, male(female)

|  | Negative | Neutral | Positive |
| --- | --- | --- | --- |
| 400 ms | 0.00(0.04) | 0.02(0.04) | 0.03(0.04) |
| 600 ms | 0.03(0.13) | 0.07(0.16) | 0.08(0.16) |
| 800 ms | 0.29(0.40) | 0.28(0.48) | 0.25(0.44) |
| 1000 ms | 0.45(0.73) | 0.59(0.71) | 0.48(0.71) |
| 1200 ms | 0.76(0.81) | 0.80(0.83) | 0.71(0.90) |
| 1400 ms | 0.86(0.91) | 0.90(0.93) | 0.87(0.91) |
| 1600 ms | 0.93(0.96) | 0.93(0.97) | 0.91(0.96) |

**Table S2** Temporal distortion effect for different genders and emotion conditions in Experiment 1(M ± SE)

|  | Negative | Neutral | Positive |
| --- | --- | --- | --- |
| Male | 18.78±43.27 | 57.91±43.27 | 5.82±43.27 |
| Female | 145.18±40.28 | 171.97±40.28 | 178.24±40.28 |

**Table S3** Weber Ratio for different genders and emotion conditions in Experiment 1(M ± SE)

|  | Negative | Neutral | Positive |
| --- | --- | --- | --- |
| Male | 0.258±0.035 | 0.259±0.035 | 0.314±0.035 |
| Female | 0.283±0.033 | 0.300±0.033 | 0.278±0.033 |

**Table S4** Mean proportion of “Long” for different conditions in Experiment 2, male(female)

|  | Low-Arousal  Negative | High-Arousal  Negative | Low-Arousal  Positive | High-Arousal  Positive | Neutral |
| --- | --- | --- | --- | --- | --- |
| 400 ms | 0.03(0.00) | 0.00(0.01) | 0.03(0.02) | 0.02(0.01) | 0.02(0.01) |
| 600 ms | 0.05(0.09) | 0.11(0.13) | 0.09(0.09) | 0.09(0.11) | 0.05(0.10) |
| 800 ms | 0.30(0.40) | 0.28(0.37) | 0.29(0.40) | 0.33(0.46) | 0.35(0.51) |
| 1000 ms | 0.69(0.81) | 0.63(0.77) | 0.61(0.73) | 0.64(0.75) | 0.63(0.75) |
| 1200 ms | 0.88(0.85) | 0.84(0.89) | 0.84(0.90) | 0.88(0.89) | 0.89(0.89) |
| 1400 ms | 0.96(0.95) | 0.99(0.90) | 0.95(0.97) | 0.95(0.95) | 0.96(0.92) |
| 1600 ms | 0.99(0.98) | 0.98(0.98) | 0.97(0.98) | 0.96(0.99) | 0.97(0.99) |

**Table S5** Temporal distortion effect for different genders and emotion conditions in Experiment 2(M ± SE)

|  | Low-Arousal  Negative | High-Arousal  Negative | Low-Arousal  Positive | High-Arousal  Positive | Neutral |
| --- | --- | --- | --- | --- | --- |
| Male | 105.30±35.55 | 83.75±35.55 | 80.35±35.55 | 104.52±35.55 | 107.69±35.55 |
| Female | 164.10±35.55 | 156.89±35.55 | 160.59±35.55 | 164.40±35.55 | 187.11±35.55 |

**Table S6** Weber Ratio for different genders and emotion conditions in Experiment 2(M ± SE)

|  | Low-Arousal  Negative | High-Arousal  Negative | Low-Arousal  Positive | High-Arousal  Positive | Neutral |
| --- | --- | --- | --- | --- | --- |
| Male | 0.191±0.023 | 0.203±0.023 | 0.216±0.023 | 0.200±0.023 | 0.213±0.023 |
| Female | 0.217±0.023 | 0.243±0.023 | 0.221±0.023 | 0.228±0.023 | 0.243±0.023 |
