# Supplementary material for: Women Overestimate Temporal Duration: Evidence from Chinese Emotional Words
Source: Front Psychol. 2017 Jan 18;8:4. doi: 10.3389/fpsyg.2017.00004 (PMC5241309; doi:10.3389/fpsyg.2017.00004)
Supplement: Supplementary file 2 [file Data_Sheet_2.docx]

**Appendix**

**Table A1** The word stimuli used in the Experiment 1

| Positive adjective | Negative adjective | Neutral adjective |
| --- | --- | --- |
| 高尚[noble] | 消沉[downhearted] | 艰巨[arduous] |
| 诚恳[hearty] | 自私[selfish] | 剧烈[acute] |
| 永恒[eternal] | 虚伪[hypocritical] | 粗壮[burly] |
| 伟大[great] | 烦恼[vexed] | 坚硬[solid] |
| 鲜艳[brilliant] | 悲观[pessimistic] | 淘气[naughty] |
| 珍贵[precious] | 悲哀[lamentable] | 稀罕[scarce] |
| 旺盛[vigorous] | 黑暗[gloomy] | 奇怪[strange] |
| 无私[selfless] | 无情[ruthless] | 凑巧[coincidental] |
| 精致[exquisite] | 失意[frustrated] | 纯粹[pure] |
| 圣洁[holy] | 丑陋[ugly] | 威严[dignified] |

**Table A2** The word stimuli used in the Experiment 2

| Low-Arousal Positive | High-Arousal Positive | Low-Arousal Negative | High-Arousal Negative | Neutral adjective |
| --- | --- | --- | --- | --- |
| 温和[mild] | 勇敢[brave] | 怯懦[timid] | 可耻[degrading] | 艰巨[arduous] |
| 悠闲[leisure] | 神奇[magical] | 苍凉[desolate] | 歹毒[vicious] | 剧烈[acute] |
| 祥和[harmonious] | 狂欢[exultant] | 困倦[sleepy] | 凶悍[fierce] | 粗壮[burly] |
| 纯净[clean] | 辉煌[glorious] | 孤单[lonely] | 淫秽[obscene] | 坚硬[solid] |
| 秀气[delicate] | 一流[topping] | 蹩脚[crappy] | 恼火[annoyed] | 淘气[naughty] |
| 安康[healthy] | 高尚[noble] | 苍白[pale] | 险恶[dangerous] | 稀罕[scarce] |
| 甘甜[sweet] | 崇高[sublime] | 贫寒[poor] | 野蛮[barbaric] | 奇怪[strange] |
| 洁白[white] | 渊博[knowledgeable] | 平庸[ordinary] | 猖狂[savage] | 凑巧[coincidental] |
| 芬芳[aromatic] | 兴奋[excited] | 暗淡[dim] | 惊恐[terrified] | 纯粹[pure] |
| 文静[quiet] | 漂亮[beautiful] | 无能[incapable] | 危急[desperate] | 威严[dignified] |
